# Comprehensive Genomics and Proteomics Analysis Reveals the Multiple Response Strategies of Endophytic *Bacillus* sp. WR13 to Iron Limitation

**DOI:** 10.3390/microorganisms11020367

**Published:** 2023-02-01

**Authors:** Zonghao Yue, Yongchuang Liu, Yanjuan Chen, Can Chen, Ju Zhang, Le He, Keshi Ma

**Affiliations:** 1 College of Life Sciences and Agronomy, Zhoukou Normal University, Zhoukou 466001, China; 2 School of Mechanical and Electrical Engineering, Zhoukou Normal University, Zhoukou 466001, China; 3 Henan Key Laboratory of Plant Molecular Breeding and Bioreactor, Zhoukou 466001, China

**Keywords:** *Bacillus*, iron limitation, siderophore, genomics, proteomics

## Abstract

Iron (Fe) is an important metal element for the growth of bacteria. Many bacteria respond to Fe limitation through a variety of strategies. We previously isolated an endophyte *Bacillus* sp. WR13 from wheat root. However, whether and how this strain can cope with Fe-deficient environments remains unclear. In this study, the growth of WR13 under Fe starvation was investigated, and the underlying mechanisms of WR13 in response to Fe starvation were elucidated via genomics and iTRAQ-based proteomics. Under Fe limitation, WR13 showed a growth pattern similar to that of Fe sufficiency. Genomics analysis demonstrated that WR13 had gene clusters related to siderophore synthesis (dhbACEBF), transportation (bcbE), uptake (feuABC-yusV) and hydrolysis (besA). These genes were significantly up-regulated in Fe-starved WR13, which resulted in more siderophore production. Proteomics data revealed that many Fe-containing proteins such as ACO, HemQ, ferredoxin, CNP, and SufD were significantly reduced under Fe limitation. Meanwhile, significant decreases in many proteins involved in glycolysis, TCA cycle, pentose phosphate pathway; asparagine, glutamine, methionine, and serine metabolism; and phospholipid hydrolysis were also observed. Overall, this study shows that *Bacillus* sp. WR13 was able to respond to Fe limitation via multiple strategies and provides a theoretical basis for the application of WR13 in Fe-deficient soil.

## 1. Introduction

Iron (Fe) is an essential trace element and has irreplaceable functions in the growth of bacteria. As a cofactor of many enzymes and proteins, it participates in many biological processes, including DNA synthesis, electron transfer, oxygen metabolism, and biofilm formation [1,2]. Although abundant in the Earth’s crust, most of Fe exists in the form of low-soluble ferric compounds, which leads to very low Fe bioavailability [3]. Therefore, Fe limitation is a great challenge for bacteria.

In order to adapt to Fe limitation and maintain Fe homeostasis, bacteria have evolved sophisticated strategies. Among them, the production of siderophores is an important way for bacteria to obtain Fe from the surrounding environment [4]. Siderophores are low-molecular-weight, high-affinity Fe chelators synthesized and secreted by many bacteria, and over 500 different types of siderophores have been discovered [5,6]. These siderophores can enhance Fe acquisition of bacteria from their surroundings through the promotion of the dissolution of Fe oxides and competition with natural Fe complexes [7,8]. In the *Bacillus* genus, Bacillibactin (BB) is the major catechol-based siderophore composed of dihydroxybenzoate, threonine, and glycine [9]. Under Fe-deficient conditions, siderophore BB is synthesized by DhbACEBF gene cluster and exported to the environment, where it chelates Fe and is then imported into cells by the FeuABC-YusV uptake system [10].

In addition to Fe acquisition, many bacteria respond to Fe limitation by repressing the synthesis of non-essential Fe-containing proteins and sparing intracellular Fe for essential cellular functions [11]. This strategy has been observed in *B. subtilis*, *Caulobacter crescentus*, *Escherichia coli*, *Pseudomonas aeruginosa*, and *Staphylococcus aureus* [12,13,14,15,16]. Notably, these alterant Fe-containing proteins in different bacteria are various under Fe deficiency. Due to the pivotal role of Fe in a series of metabolic pathways, the strategy may result in the remodeling of bacterial metabolic networks.

In a previous study, we isolated a series of endophytes including *Bacillus* sp. WR13 from wheat root [17]. However, whether this strain can grow under Fe limitation conditions and how it adapts to Fe starvation are still unknown. Herein, the growth of WR13 was first evaluated under different Fe conditions. Then, the molecular mechanisms of WR13 in response to Fe limitation were explored at the genetic and protein levels by using genomics and iTRAQ-labeled quantitative proteomics. These results would further increase the current understanding of *Bacillus* sp.’s response to Fe starvation.

## 2. Materials and Methods

### 2.1. Bacteria Growth and Treatment

Strain *Bacillus* sp. WR13 was inoculated into Luria–Bertani (LB) broth and cultured overnight at 37 °C at 150 rpm. Then, bacterial suspension (1%, *v*/*v*) was transferred into 100 mL of LB broth with or without 100 μM 2,2′-dipyridyl (Sigma-Aldrich, Saint Louis, MO, USA), an Fe chelator often used to create Fe-deficient conditions in a medium [18,19]. Each group contained three replications. After 24 h, the bacterial suspension from each group was divided into two equal parts. One part was used to evaluate the bacterial growth. Specifically, 200 μL bacterial suspension from each replication was transferred to a 96-well microplate, and the OD600 was measured using a SpectraMax i3x microplate reader (Molecular Devices, Sunnyvale, CA, USA). The remaining bacterial suspension was centrifuged at 12,000 rpm for 5 min, and the bacterial cells were collected and weighed after drying at 60 °C for 48 h. The other part was centrifuged under the same conditions, and the supernatants and bacterial cells were collected, respectively. The supernatants were used to determine the siderophore. The cells were rinsed with sterile water, frozen in liquid nitrogen, and stored at −80 °C for real-time quantitative PCR (qPCR) and proteomics analysis.

### 2.2. Draft Genome Sequencing of WR13

According to the above method, WR13 was incubated in LB broth and collected. The extraction of genomic DNA and DNA concentration and integrity analysis were carried out according to our previous methods [20]. High-quality genomic DNA was sheared into 400–500 bp fragments using a Covaris M220 Focused Acoustic Shearer (Woburn, MA, USA). These fragments were then used to construct Illumina sequencing libraries using the NEXTflex™ Rapid DNA-Seq Kit (PerkinElmer, Waltham, MA, USA). Draft genome sequencing was performed on an Illumina Hiseq X ten system (Illumina, San Diego, CA, USA) by Majorbio Bio-Pharm Technology Co., Ltd. (Shanghai, China).

After sequencing, the raw reads were firstly filtered using fastp software (version 0.19.6) to obtain high-quality clean reads. Then, these clean reads were further assembled into scaffolds using SOPA de novo version 2.04. Glimmer v3.02, tRNA-scan-SE v2.0 and Barrnap software were used to predict coding sequences (CDSs), tRNA and rRNA in the draft genome, respectively. Functional annotation was conducted by BLASTP comparisons of the predicted CDSs with the NR, Swiss-Prot, Pfam, GO, COG and KEGG databases (e-value < 10^−5^). In addition, the prediction of secondary metabolite biosynthetic gene clusters (SM-BGCs) was performed using antiSMASH software. The draft genomic data of WR13 were submitted to GenBank with the accession number JANSKD000000000 and further annotated automatically by the NCBI prokaryotic genome annotation pipeline.

### 2.3. Siderophore Determination

The determination of the siderophore was performed according to a previous method [21]. Briefly, 1.0 mL of supernatant was mixed sequentially with 125 μL of 10 mM FeCl_3_ (dissolved in 100 mM HCl) and 100 μL of 1 M Tris-HCl (pH 8.0). The absorbance was determined at 510 nm using a SpectraMax i3x microplate reader. The standard curve was prepared with 3,4-dihydroxybenzoic acid (3,4-DHBA, Sigma-Aldrich). The siderophore concentration is presented as μg/mL DHBA/OD600.

### 2.4. RNA Isolation and qPCR Assay

The total RNA of WR13 was isolated using a bacteria RNA extraction kit (Vazyme, Nanjing, China). Then, the RNA concentration and quality were evaluated by using a Nanodrop2000c ultra-micro spectrophotometer (ThermoFisher, Waltham, MA, USA). A measure of 500 ng of RNA was subjected to cDNA synthesis using a HiScript III RT SuperMix for qPCR (+gDNA wiper) kit (Vazyme, Nanjing, China). qPCR amplification was then performed using a ChamQ SYBR qPCR Master Mix kit (Vazyme, Nanjing, China) in a CFX96 Touch Real-Time PCR Detection System (Bio-Rad, Hercules, CA, USA). The relative mRNA expressions of siderophore-related genes were normalized with the housekeeping gene 50S ribosomal protein L2 (rpLB) and calculated using the 2^−ΔΔCt^ method. Primers of these target genes were listed in Supplementary Table S1.

### 2.5. Total Protein Extraction, Digestion and iTRAQ Labeling

The bacteria cell samples were homogenized with lysis buffer (8 M urea and 1% sodium deoxycholate) using a tissue grinder (Wonbio, Shanghai, China). The homogenates were placed on ice for 30 min, during which the samples were vortexed every 5 min. After that, the mixtures were centrifugated for 30 min at 16,000× *g* at 4 °C, and the supernatants containing protein were collected. The protein concentration was measured using a Pierce BCA Protein Assay Kit (Thermo Fisher, Waltham, MA, USA). In addition, SDS-PAGE was performed for the determination of protein quality by using 10% Bis-Tris NuPAGE gels (Invitrogen, Carlsbad, CA, USA).

The sample containing 100 μg of total protein was mixed with 100 μL of the lysate and then Bond-Breaker TCEP solution (final concentration of 10 mM, Thermo Fisher Scientific) was added. After incubation at 37 °C for 1 h, iodoacetamide (final concentration of 40 mM, Sigma-Aldrich) was added. The tubes were placed in the dark at room temperature for 40 min and precooled acetone was added in the ratio of 6:1. These samples were precipitated at −20 °C for 4 h and centrifuged at 10,000× *g* for 20 min at 4 °C. The collected protein precipitation was resuspended in 100 µL of 50 mM triethylammonium bicarbonate buffer (TEAB, Sigma-Aldrich). The proteins were then digested with modified trypsin (Promega, Madison, WI, USA) at a 1:50 enzyme-to-substrate mass ratio. After 37 °C overnight, the resulting peptide mixtures were labeled using iTRAQ reagent from the iTRAQ Reagent 8Plex Multi-Plex Kit (AB Sciex, Framingham, MA, USA) for 2 h at room temperature. Subsequently, 50 µL of ultrapure water was added and stored at room temperature for 30 min. Finally, all samples were pooled, desalted, and vacuum-dried.

### 2.6. High pH Reverse Phase Separation and LC-MS/MS Analysis

The pooled mixtures of iTRAQ-labeled peptides were redissolved in 2.0% acetonitrile (pH 10.0) and then fractionated by a Vanquish Flex ultra-performance liquid chromatography system (Thermo Fisher Scientific) with ACQUITY UPLC BEH C18 Column (1.7 µm, 2.1 mm × 150 mm, Waters, Milford, CT, USA). The mobile phase contained 2.0% acetonitrile (solvent A) and 80.0% acetonitrile (solvent B). Specifically, the peptides were eluted at a flowrate of 200 μL/min using the following linear gradient: 100% A for 16 min; 0~3.8% B for 1 min; 3.8%~24% B for 17 min; 24%~30% B for 3 min; 30%~43% B for 1 min; 43%~100% B for 1 min; 100% B for 4 min; 100%~0% B for 1 min; 100% A for 3 min. Twenty fractions were collected from each sample, which was subsequently pooled into ten final fractions per sample and dried by vacuum centrifugation.

After redissolved with 2.0% acetonitrile containing 0.1% formic acid, the fractions were analyzed by online nanoflow liquid chromatography tandem mass spectrometry performed on an EASY-nLC 1200 system connected to a Q-Exactive HF-X mass spectrometer (Thermo Fisher Scientific). First, the C18 column (75 μm × 25 cm, Thermo Fisher Scientific) was equilibrated with solvent A (2.0% acetonitrile with 0.1% formic acid) and solvent B (80.0% acetonitrile with 0.1% formic acid). Next, the peptides were eluted at a flowrate of 300 nL/min using the following gradient: 5~23% B for 63 min; 23%~29% B for 19 min; 29%~38% B for 8 min; 38%~48% B for 2 min; 48%~100% B for 2 min; 100% B for 28 min. Later, the peptide ions were analyzed using the Q-Exactive HF-X, which was operated in the data-dependent acquisition mode to automatically switch between full scan MS and MS/MS acquisition. The MS scan range was 350–1300 *m*/*z* and the resolution was 60,000. The automatic gain control (AGC) target was 3e6 and the maximum fill time was 20 ms. The MS/MS scan was set at a resolution of 15,000 (at *m*/*z* 100), AGC target value of 5e4, maximum fill time of 45 ms, and dynamic exclusion time of 20 s.

### 2.7. Protein Identification and Differentially Abundant Protein (DAP) Screening

The raw data were searched against the UniProt database by using the Proteome Discoverer (version 2.4). The search criteria of protein identification were as follows: the digesting enzyme was trypsin with 2 max missed cleavage allowed. Precursor mass tolerance and fragment mass tolerance were 20 ppm and 0.05 Da, respectively. Fixed modification was carbamidomethyl (C), iTRAQ8plex (N-Terminus) and iTRAQ8plex (K). The oxidation of methionine (Met), acetyl of protein N-term, Met-loss of protein N-term were allowed as dynamic modifications. The false discovery rate (FDR) of peptide identification was set as FDR ≤ 0.01. A minimum of one unique peptide identification was used to support protein identification. The threshold of fold change (≥1.2 or ≤0.83) and *p*-value < 0.05 was used to identify DAPs. Functional annotation of these DAPs was performed by using Gene Ontology (GO), clusters of Orthologous Groups (COG) and Kyoto Encyclopedia of Genes and Genomes (KEGG) analysis. The proteomics data have been deposited to the ProteomeXchange Consortium via the iProX partner repository with the dataset identifier PXD030851.

### 2.8. Statistical Analysis

Data are expressed as mean ± standard deviation (SD). The significant differences in growth or siderophore levels between two groups were analyzed by Student’s *t*-test after conducting tests of normality (Kolmogorov–Smirnov) and equal variance (Levene’s median). All statistical analysis was performed in IBM SPSS Statistics 19.0 (IBM, Armonk, NY, USA). A *p*-value < 0.05 was considered as statistically significant.

## 3. Results

### 3.1. The Growth of WR13 under Different Fe Conditions

**Figure 1:**
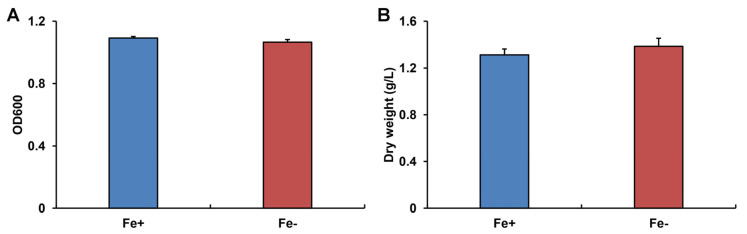
The absorbance (**A**) and dry weight (**B**) of *Bacillus* sp. WR13 after 24 h incubation in Fe-sufficient (Fe+) and Fe-deficient (Fe−) conditions.

Under Fe deficiency, WR13 showed similar growth as the control group. Specifically, there were no significant differences in OD600 (*p* = 0.081) and dry weight (*p* = 0.099) of WR13 under Fe deficiency compared with those of the control group (Figure 1A).

### 3.2. Genome Analysis of WR13

**Figure 2:**
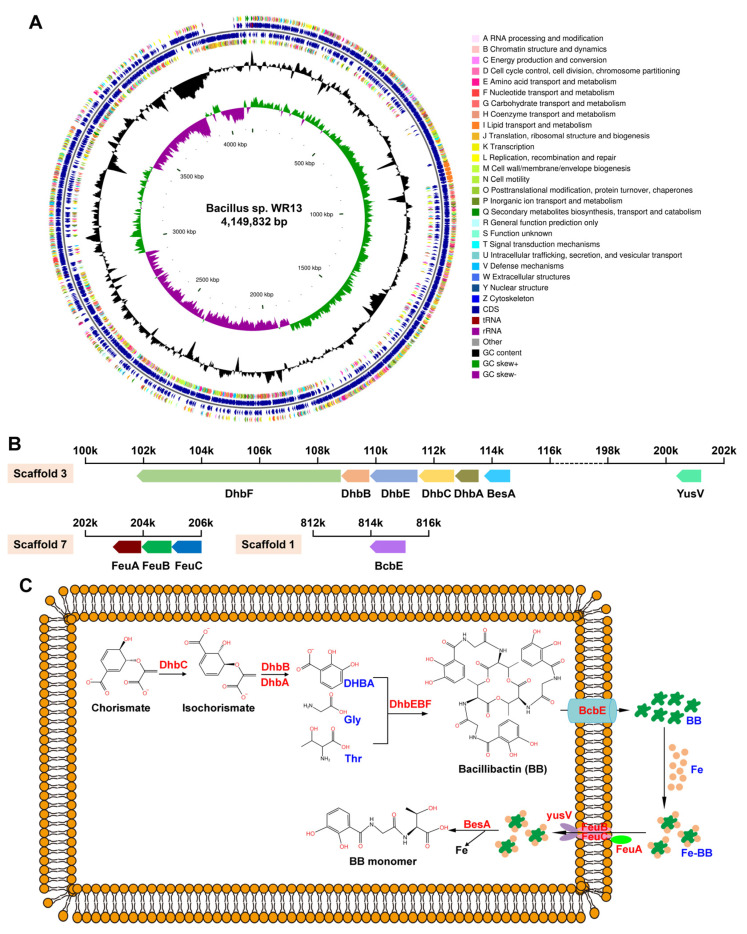
Genome information of *Bacillus* sp. WR13. (**A**) Circular genomic map of WR13. The circular map was generated using CGView software. Circles (from outside to inside) 1 and 4: CDSs on the forward and reverse strand; Circles 2 and 3: CDSs, tRNA, and rRNA on the forward and reverse strand; Circle 5: GC content; and Circles 6: GC skew. (**B**) Siderophore-related gene clusters in the chromosome. (**C**) The pathway of siderophore synthesis, transportation, uptake, and hydrolysis.

By draft genome sequencing of WR13, a total of 3,506,381 clean pair reads was obtained and assembled into a final genome size of 4,149,832 bp (4.15 Mbp) with a GC content of 43.4% (Figure 2A). The genome consists of 25 scaffolds, of which 14 scaffolds belong to chromosome with a total of 4,009,084 bp (4.01 Mbp), and 11 scaffolds belong to plasmid with a total of 140,748 bp (0.14 Mbp) (Table S2). In the chromosome, a total of 4094 CDSs, 88 Pseudo genes, 4 rRNAs, 40 tRNAs, and 5 ncRNAs were identified. The detailed genomic overview of WR13 is shown in Table S3. AntiSMASH and BLAST bioinformatics analyses identified 13 SM-BGCs. Among them, siderophore BGC (DhbACEBF) was found in scaffold 3 and contained 5 genes: dhbA, dhbC, dhbE, dhbB and dhbF (Figure 2B). In addition, bcbE, involved in BB secretion; feuA, feuB, feuC and yusV, involved in Fe-BB uptake; and besA, involved in Fe-BB hydrolysis were also found in scaffold 1, scaffold 3, and scaffold 7, respectively (Figure 2B). The pathway related to siderophore biosynthesis, secretion, uptake, and hydrolysis is shown in Figure 2C.

### 3.3. Siderophore Production and Siderophore-Related Genes Expression in WR13 under Fe Limitation

**Figure 3:**
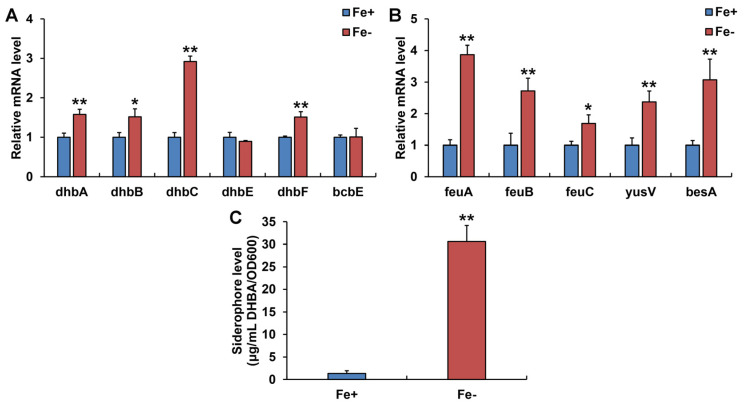
Siderophore-related gene expression and siderophore production in *Bacillus* sp. WR13 under Fe-sufficient (Fe+) and Fe-deficient (Fe−) conditions. (**A**) Gene expression related to siderophore synthesis and transportation; (**B**) gene expression related to Fe-BB uptake and hydrolysis. (**C**) Quantitative analysis of siderophore. Data are expressed as the means ± SD (*n* = 3). * *p* < 0.05 and ** *p* < 0.01 indicate significant and extremely significant differences.

At the transcription level, Fe limitation induced significant upregulation of *dhbA*, *dhbB*, *dhbC*, *dhbF*, *feuA*, *feuB*, *feuC*, *yusV*, and *besA* genes in WR13 (Figure 3A,B). Further biochemical experimentation showed that WR13 produced siderophores and the number of siderophores increased sharply 22.8-fold under Fe limitation compared with the control group (Figure 3C).

### 3.4. Quantification of Total Proteins and Protein Differential Analysis by iTRAQ-Labeled Proteomics

By proteomic analysis, a total of 3921 spectra, 747 peptides, and 463 proteins were identified. Among them, 96.52% of the peptides’ lengths were between 6 and 19, and 97.62% of the proteins’ molecular weights were between 1 and 161 kDa (Figures S1 and S2). In addition, 85.53%, 84.23%, 92.22%, and 93.30% of these proteins were annotated to GO, KEGG, GOG, and Pfam databases, respectively (Table S4).

**Figure 4:**
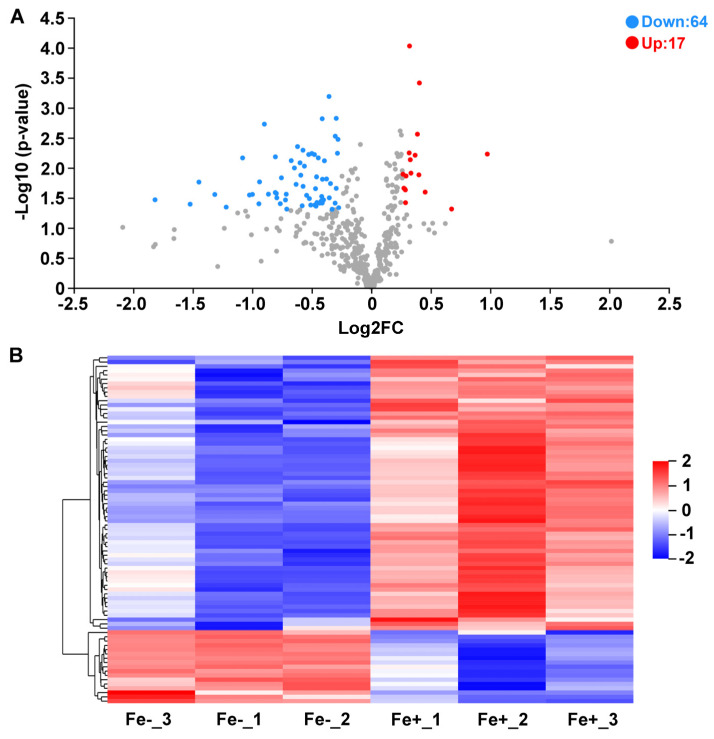
Differentially expressed proteins (DAPs) between Fe-sufficient (Fe+) and Fe-deficient (Fe−) groups. (**A**) Volcano plot of DAPs. Red dot represents up-regulated proteins and blue dot represents down-regulated proteins. (**B**) Hierarchically-clustered heatmap diagram of DAPs. Each row represents one significant protein. Fe-_1, Fe-_2, Fe-_3: three biological replicates of Fe− group; Fe+_1, Fe+_2, Fe+_3: three biological replicates of Fe+ group.

As illustrated by the volcano plot and hierarchically clustered heatmap, a total of 81 proteins were differentially expressed between Fe-sufficient and Fe-deficient groups. Among these DAPs, 17 proteins were significantly upregulated and 64 proteins were significantly downregulated in Fe-starved WR13 (Figure 4).

### 3.5. Functional Annotation of DAPs

**Figure 5:**
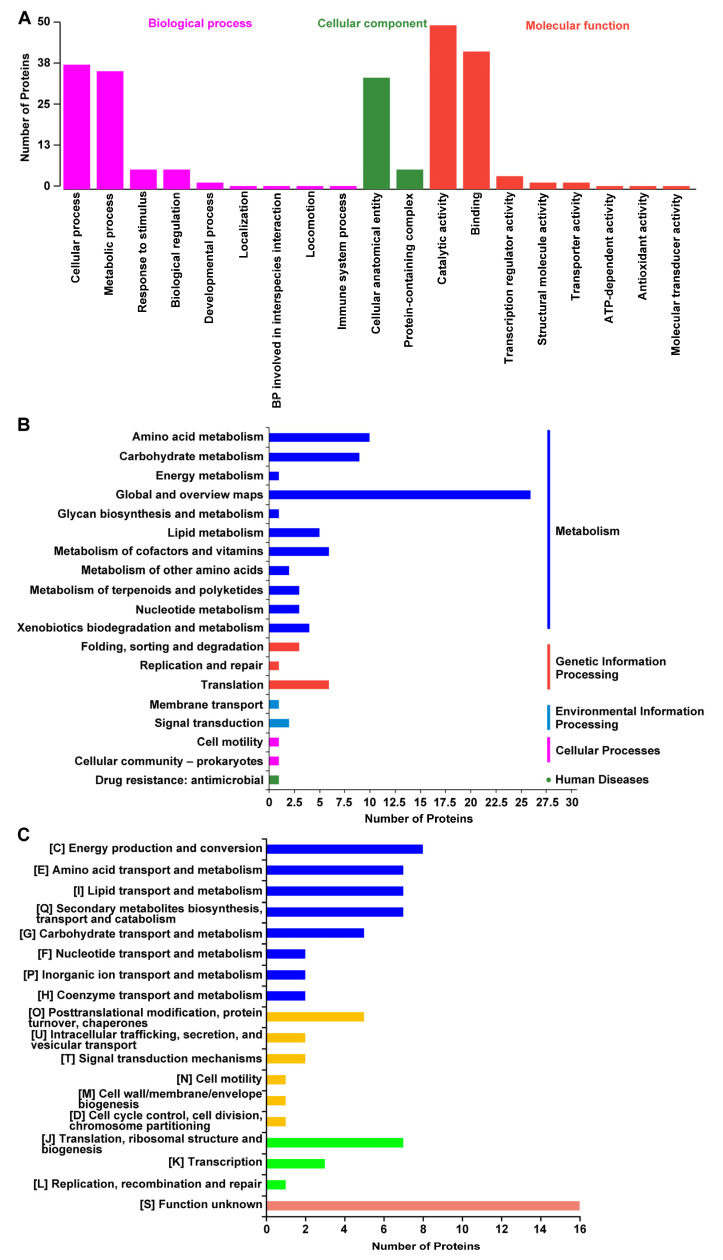
Function annotation results of DAPs in GO, KEGG and COG databases. (**A**) DAPs annotated in GO database. (**B**) DAPs annotated in KEGG pathway database. (**C**) DAPs annotated in COG database.

GO classification showed that 66 DAPs were categorized into three categories: biological process (BP), cellular component (CC), and molecular function (MF) (Figure 5A). In the BP category, DAPs mainly participated in cellular process, metabolic process, and response to stimuli. In the CC category, DAPs were assigned to the cellular anatomical entity and protein-containing complex. In the MF category, DAPs mainly performed the functions of catalytic activity and binding. KEGG annotation analysis revealed that 39 DAPs were annotated to five pathway categories, including metabolism, genetic information processing, environmental information processing, cellular processes, and diseases. Among them, metabolism was the largest category. In this category, the most represented KEGG pathway was “Global and overview maps”, followed by “Amino acid metabolism”, “Carbohydrate metabolism”, “Lipid metabolism”, and “Metabolism of cofactors and vitamins” (Figure 5B). In addition, by performing COG annotation, a total of 73 DAPs were classified into 18 categories. Among these categories, approximately half were related to metabolism, including “Amino acid transport and metabolism”, “Lipid transport and metabolism”, “Secondary metabolites biosynthesis, transport and catabolism”, and “Carbohydrate transport and metabolism” (Figure 5C). Therefore, according to GO, KEGG and COG analysis results, these DAPs were closely related to carbohydrate metabolism, amino acid metabolism and lipid metabolism.

### 3.6. The Key DAPs Involved in Carbohydrate Metabolism, Amino Acid Metabolism, Lipid Metabolism, and Fe-Containing Proteins

**Figure 6:**
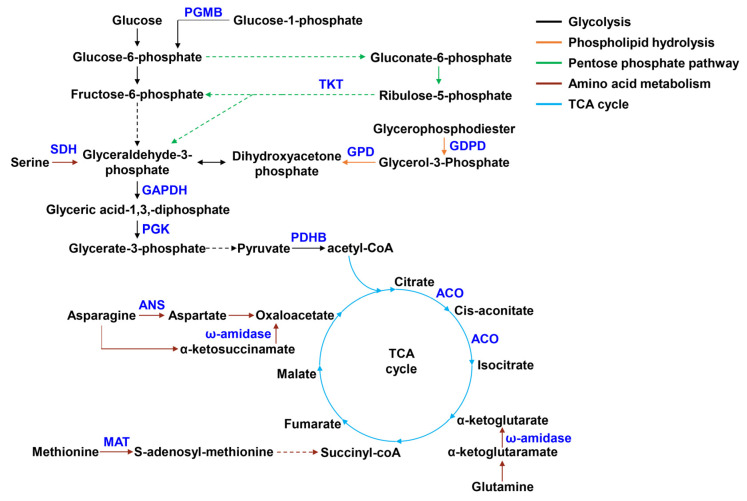
The key DAPs involved in carbohydrate, amino acid, and lipid metabolism. Blue font represents down-regulated DAPs. Different colored lines represent different metabolic pathways.

As shown in Table 1 and Figure 6, all DAPs were significantly down-regulated in carbohydrate metabolism. These DAPs included pyruvate dehydrogenase E1 component beta subunit (PDHB) and aconitase (ACO) involved in citrate cycle (TCA cycle), type I glyceraldehyde-3-phosphate dehydrogenase (GAPDH) and phosphoglycerate kinase (PGK) involved in glycolysis, transketolase (TKT) involved in pentose phosphate pathway, and beta-phosphoglucomutase (PGMB). In amino acid metabolism, DAPs related to asparagine, glutamine, methionine and serine metabolism were significantly reduced, such as asparaginase (ANS), ω-amidase, methionine adenosyltransferase (MAT), and L-serine ammonia-lyase (SDH). In lipid metabolism, glycerophosphodiester phosphodiesterase (GDPD) and glycerol-3-phosphate dehydrogenase (GPD) involved in phospholipid metabolism were significantly down-regulated. Among the above-mentioned DAPs, we noticed that ACO belonged to Fe-containing proteins. In addition to this protein, other reduced Fe-containing proteins, such as heme-dependent peroxidase HemQ, ferredoxin, 2′,3′-cyclic-nucleotide 2′-phosphodiesterase (CNP) and Fe-S cluster assembly protein SufD, were also observed.

## 4. Discussion

In a natural environment, Fe bioavailability is very low and the solubility of ferric Fe is only 10^−8^–10^−9^ M at neutral pH, which is far below the level of 10^−6^ M for optimal bacterial growth [22]. As a kind of Gram-positive bacteria widely distributed in environments, many members of the genus *Bacillus* can still survive under Fe limitation conditions. In this study, *Bacillus* sp. WR13, a wheat endophyte, was also found to grow well under Fe deficiency. Similar results were also observed in *B. subtilis*, *B. megaterium*, and *B. anthracis* [23,24,25].

It is well known that many microorganisms respond to Fe starvation by producing a low-molecular-weight compounds called siderophores [26]. As the Fe-chelating agents, siderophores can capture insoluble Fe^3+^ from surroundings [27]. In *Bacillus* strains, BB is the major catechol-based siderophore synthesized by DhbACEBF gene cluster [28]. After synthesis, the mature BB is secreted into the extracellular matrix by YmfD (or BcbE) to chelate Fe [29]. The Fe-BB complex is then transported into the cell by the feuABC-encoded ABC transporter and yusV-encoded ATPase, and hydrolyzed by besA-encoded trilactone hydrolase to release Fe [24,30]. By genome analysis, we also found that WR13 had these genes related to BB synthesis, transportation, uptake, and hydrolysis, indicating that WR13 may produce siderophores. qPCR results showed that Fe limitation significantly up-regulated *dhbA*, *dhbB*, *dhbC*, and *dhbF*, involved in BB synthesis; *feuA*, *feuB*, *feuC*, and *yusV*, involved in Fe-BB uptake; and besA, involved in BB hydrolysis in WR13. This indicated that Fe-starved WR13 had more siderophore production. A subsequent biochemical test confirmed this. Therefore, we believe that improving Fe acquisition by producing more siderophores is one of the important strategies of WR13 to cope with Fe limitation.

To further explore the molecular mechanism of WR13 in response to Fe limitation, the proteomics of WR13 were performed. Many Fe-containing proteins, such as ACO, HemQ, ferredoxin, CNP, and SufD, were significantly down-regulated under Fe-starved WR13. It is reported that when Fe deprivation occurs, many bacteria reduce the demand for Fe-containing proteins and preserve Fe for vital processes [31]. This phenomenon has been described in many bacteria. For example, *B. subtilis* downregulated Fe-containing enzymes ACO, glutamate synthase, succinate dehydrogenase, and C4-dicarboxylate permease under Fe-limiting conditions [32]. Fe starvation resulted in a decrease in iron-cofactored superoxide dismutase SodB, heme-cofactored catalase KatA, and iron storage protein bacterioferritin B in *Pseudomonas aeruginosa* [33]. Therefore, we suggested that the inhibition of Fe-containing proteins ACO, HemQ, ferredoxin, CNP and SufD may be another effective strategy for WR13 to respond to Fe starvation.

Further functional annotation showed that many DAPs were related to carbohydrate, amino acid, and lipid metabolism. In carbohydrate metabolism, all related proteins (GAPDH, PGK, PDHB, ACO, TKT) were significantly down-regulated under Fe limitation. GAPDH and PGK are key enzymes that catalyze the conversion of glyceraldehyde 3-phosphate to 3-phosphoglycerate in glycolysis [34]. PDH participates in the conversion of pyruvate produced by glycolysis to acetyl-CoA, the initial substance of the TCA cycle. ACO catalyzes the production of isocitrate from citrate in the TCA cycle [35]. Transketolase (TKT) is a key enzyme in the pentose phosphate (PP) pathway and plays a critical role in the production of fructose-6-phosphate and glyceraldehyde 3-phosphate [36]. Therefore, a decrease in these DAPs indicated that WR13 inhibited glycolysis, TCA cycle and PP pathway under Fe limitation. In amino acid metabolism, ANS, ω-Amidase, MAT and SDH were significantly reduced in Fe-starved WR13. ANS is an enzyme that catalyzes the hydrolysis of L-asparagine to aspartate [37]. ω-Amidase hydrolyses α-ketoglutaramate to α-ketoglutarate in L-glutamine metabolism and α-ketosuccinamate to oxaloacetate in asparagine metabolism [38,39]. MAT and SDH catalyzes the conversion of L-methionine to S-adenosyl methionine and L-serine to pyruvate, respectively [40,41]. Hence, the reduction of these enzymes induced by Fe limitation demonstrated that asparagine, glutamine, methionine, and serine metabolism was inhibited. Together, these results suggested that Fe limitation inhibited carbohydrate and amino acid metabolism of WR13. Significant changes in carbohydrate metabolism and amino acid metabolism were also observed in nutrient-poor *E. coli* [42]. Therefore, WR13 may adjust its metabolism in response to environmental Fe deprivation.

In addition, decreased GDPD and GPD related to lipid metabolism were also observed under Fe limitation. GDPD is a key enzyme in the phospholipid hydrolysis process and degrades glycerophosphodiester to glycerol-3-phosphate (G3P) [43]. G3P is then catalyzed by GPD to produce dihydroxyacetone phosphate, an intermediate of glycolysis [44]. Therefore, a reduction in these two enzymes may result in the inhibition of phospholipid hydrolysis, thereby causing phospholipid accumulation. Considering that phospholipid is an important component of the cell membrane, we suggested that WR13 may cope with Fe limitation by improving the cell membrane integrity.

## 5. Conclusions

In summary, our study showed that *Bacillus* sp. WR13 was a well-grown strain under Fe limitation. Genomics and proteomics data revealed that WR13 coped with Fe limitation by several different strategies: (1) increasing Fe acquisition by activating genes related to siderophore synthesis, uptake, and hydrolysis and promoting siderophore production; (2) reducing Fe-containing proteins; (3) inhibiting carbohydrate and amino acid metabolism; and (4) improving cell membrane integrity by the inhibition of phospholipid hydrolysis. Considering that WR13 is a wheat endophyte and many members of *Bacillus* have been proven to alleviate Fe deficiency in crops [20,45], the results in this study will be helpful to future research on the application of WR13 in Fe deficiency stress of crops.

## Figures and Tables

**Table 1:** The key DAPs in response to Fe limitation.

Accession	Description	FC (Fe−/Fe+)	*p* Value	Regulate
**Carbohydrate metabolism**				
gene1651	pyruvate dehydrogenase E1 component beta subunit (PDHB)	0.718	0.006	down
gene4021	aconitase (ACO)	0.747	0.036	down
gene2203	phosphoglycerate kinase (PGK)	0.749	0.030	down
gene2204	type I glyceraldehyde-3-phosphate dehydrogenase (GAPDH)	0.660	0.008	down
gene4010	transketolase (TKT)	0.367	0.017	down
gene2263	beta-phosphoglucomutase (PGMB)	0.501	0.028	down
**Amino acid metabolism**				
gene1700	Asparaginase (ANS)	0.572	0.007	down
gene0508	ω-amidase	0.816	0.022	down
gene0981	methionine adenosyltransferase (MAT)	0.723	0.022	down
gene0743	L-serine ammonia-lyase, iron-sulfur-dependent subunit beta (SDH)	0.729	0.038	down
**Lipid metabolism**				
gene3591	glycerophosphodiester phosphodiesterase (GDPD)	0.810	0.039	down
gene0063	glycerol-3-phosphate dehydrogenase (GPD)	0.725	0.044	down
**Fe-containing proteins**				
gene2592	heme-dependent peroxidase HemQ	0.549	0.028	down
gene1752	ferredoxin	0.348	0.040	down
gene0858	2′,3′-cyclic-nucleotide 2′-phosphodiesterase (CNP)	0.537	0.002	down
gene2067	Fe-S cluster assembly protein SufD	0.741	0.039	down

## Data Availability

The annotated draft genome sequence of the WR13 is available at GenBank under accession number JANSKD000000000. The proteomics data reported herein are available at the ProteomeXchange Consortium with the dataset identifier PXD030851. Data will be made available on request.

## Supplementary Materials

The following supporting information can be downloaded at: https://www.mdpi.com/article/10.3390/microorganisms11020367/s1, Figure S1: Distribution of peptide length of the identified proteins in WR13 grown under Fe-sufficient (Fe+) and Fe-deficient (Fe−) conditions; Figure S2: Distribution of molecular weight of the identified proteins in WR13 grown under Fe-sufficient (Fe+) and Fe-deficient (Fe−) conditions; Table S1: The primer sequences for qPCR assay in *Bacillus* sp. WR13; Table S2: The assembled results of *Bacillus* sp. WR13 genome; Table S3: Genome statistical information for *Bacillus* sp. WR13; Table S4: Summary of the protein annotations in four databases.
