# Supplementary material for: Comprehensive Genomics and Proteomics Analysis Reveals the Multiple Response Strategies of Endophytic Bacillus sp. WR13 to Iron Limitation
Source: Microorganisms. 2023 Feb 1;11(2):367. doi: 10.3390/microorganisms11020367 (PMC9961900; doi:10.3390/microorganisms11020367)
Supplement: Supplementary file 1 [file microorganisms-11-00367-s001.zip › microorganisms-2192734-supplementary.pdf]

Supplementary Materials

Comprehensive Genomics and Proteomics Analysis Reveals the Multiple Response Strategies of Endophytic *Bacillus* sp. WR13 to iron limitation

Zonghao Yue [1], Yongchuang Liu [1], Yanjuan Chen [2], Can Chen [3], Ju Zhang [3], Le He [1], Keshi Ma [1,*]

[1] College of Life Sciences and Agronomy, Zhoukou Normal University, Zhoukou 466001, China

[2] School of Mechanical and Electrical Engineering, Zhoukou Normal University, Zhoukou 466001, China

[3] Henan Key Laboratory of Plant Molecular Breeding and Bioreactor, Zhoukou 466001, China

* Correspondence: zknumks@163.com (K.M.); Tel.: +86-15896720176

Supplementary tables

Table S1 The primer sequences for qPCR assay in *Bacillus* sp. WR13

Gene number	Gene name	Forword primer sequence (5′–3′)	Reverse primer sequence (5′–3′)	Product length (bp)
Gene1997	DhbA	ATTGTATCTCCCGGATCAACGG	TCCTTTTATGACTTGCTCCGCT	84
Gene1994	DhbB	CAAATGGCGCTGGACTATGC	TTTGCCAGTGTTTGCTGACG	120
Gene1996	DhbC	ATCGCTGCATTTGACATCGC	CAAACGTGTAGCCATGCGAG	89
Gene1995	DhbE	AGTCCAGATGACGCATTTCCTT	TCCATCCATACCATCGCAAGAG	86
Gene1993	DhbF	CACCAAACGGGATGGAAGGA	CGCATCAATCGGTCAGCAAG	88
Gene3634	FeuA	AGACGCGAAATTGCTTGACG	TCTCCGGTTGGTTTGGCTTT	105
Gene3635	FeuB	AATTGCGTTTGTCGGGTTGG	CTTGCGAGATCACACAGGGT	126
Gene3636	FeuC	AGCGGTTTAATCGGGATGCT	ATCGCCAAAATGATGCCTGC	98
Gene2093	YusV	TATCATCCTTCTTGACGAGCCG	TCGTCCGGTCTTCCTTTTCATT	101
Gene0846	BcbE	GAAAGTGAACAATGCGGAACCA	GCAAAACGCCGAGAGTATGAAA	135
Gene1998	BesA	AAAAGCCGGATGAACGACAATG	GGCTGACCAAAACAGGAAGAAC	133
Gene4093	RplB	TCTGCTTGCCGTGCTTCTAT	ACCGTGTGGGTGATCGTTAG	144

Table S2 The assembled results of *Bacillus* sp. WR13 genome

Scaffold ID	Length (bp)	GC content (%)	Sequence Type
Scaffold1	961598	43.48	Chromosome
Scaffold2	890688	43.25	Chromosome
Scaffold3	704946	43.96	Chromosome
Scaffold4	362198	44.41	Chromosome
Scaffold5	285834	45.33	Chromosome
Scaffold6	224470	43.23	Chromosome
Scaffold7	209767	45.05	Chromosome
Scaffold8	159218	40.98	Chromosome
Scaffold10	64989	42.16	Chromosome
Scaffold11	59668	42.33	Chromosome
Scaffold12	55306	42.62	Chromosome
Scaffold13	15806	41.78	Chromosome
Scaffold15	10388	45.93	Chromosome
Scaffold17	4208	53.27	Chromosome
Scaffold16	4903	34.18	Plasmid
Scaffold18	707	57.99	Plasmid
Scaffold20	557	55.66	Plasmid
Scaffold9	118711	34.85	Plasmid
Scaffold14	13104	44.73	Plasmid
Scaffold19	657	49.92	Plasmid
Scaffold21	515	52.23	Plasmid
Scaffold22	509	58.35	Plasmid
Scaffold23	460	55.77	Plasmid
Scaffold24	325	56	Plasmid
Scaffold25	300	45.67	Plasmid

Table S3 Genome statistical information for *Bacillus* sp. WR13.

**Attribute**	**Value**
Total Genome Size (bp)	4,149,832
Raw pair reads	3,567,158
Clean pair reads	3,506,381
Clean Data Q20 (%)	96.82
GC Content (%)	43.4
Chromosome	
Chromosome Size (bp)	4,009,084
Number of Scaffolds	14
CDs Number	4,094
Pseudo genes	88
rRNA genes	4
tRNA genes	40
ncRNA genes	5

Table S4 Summary of the protein annotations in four databases

**Data base**	**Protein number**	**Percentage**
GO	396	85.53%
KEGG	390	84.23%
COG	427	92.22%
Pfam	432	93.30%
Total annotations	463	100.00%

Supplementary figures

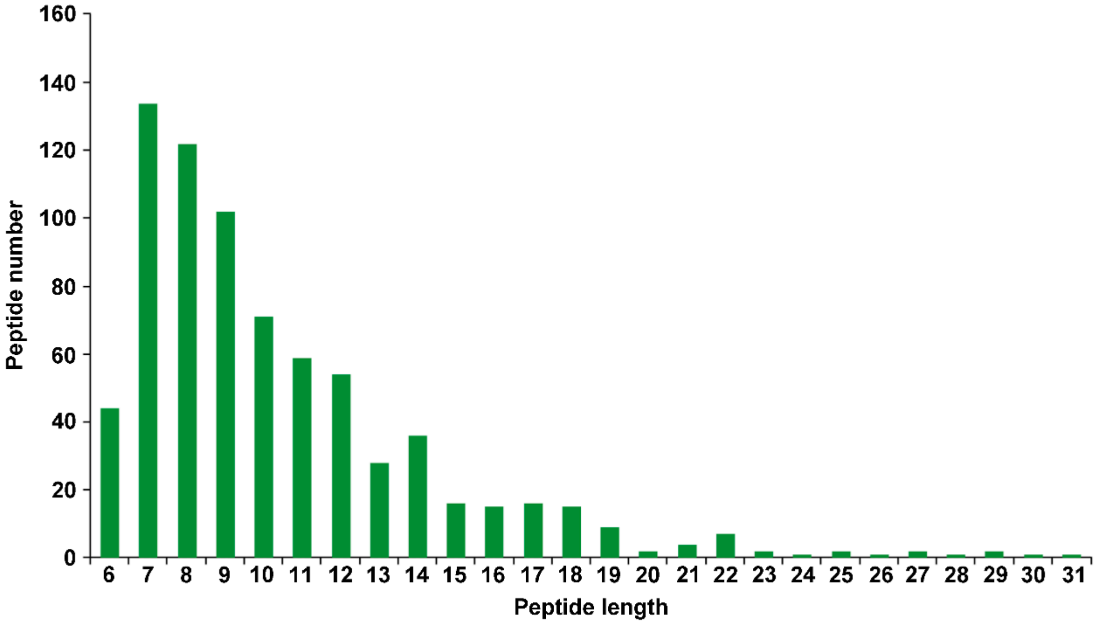

Figure. S1 Distribution of peptide length of the identified proteins in WR13 grown under Fe-sufficient (Fe+) and Fe-deficient (Fe-) conditions.

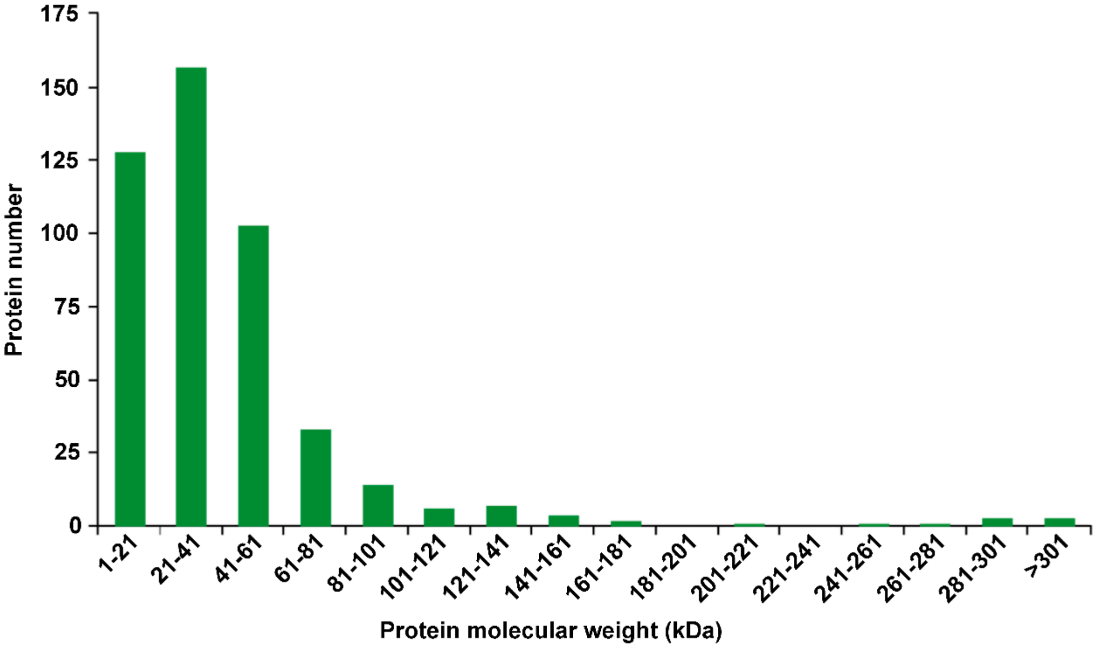

Figure. S2 Distribution of molecular weight of the identified proteins in WR13 grown under Fe-sufficient (Fe+) and Fe-deficient (Fe-) conditions.
